# Entactogens: How the Name for a Novel Class of Psychoactive Agents Originated

**DOI:** 10.3389/fpsyt.2022.863088

**Published:** 2022-03-25

**Authors:** David E. Nichols

**Affiliations:** Division of Chemical Biology and Medicinal Chemistry, The University of North Carolina at Chapel Hill, Chapel Hill, NC, United States

**Keywords:** entactogen, MDMA, MBDB, serotonin, serotonin transporter

## Abstract

At first glance, it appears there is little difference between the molecular structures of methylenedioxymethamphetamine (MDMA), which has an *N*-methyl attached to its amino group, and methylenedioxyamphetamine (MDA), a primary amine that is recognized to have hallucinogenic activity. It is known from studies with other hallucinogenic amphetamines that *N*-methylation of hallucinogenic amphetamines attenuates or abolishes hallucinogenic activity. Nevertheless, MDMA is biologically active and has a potency only slightly less than its MDA parent. Importantly, it is the Ievo-isomer of hallucinogenic phenethylamines that is more biologically active, whereas it is the dextro isomer of MDMA that is more active. This reversal of stereochemistry for the activity of two very closely related molecules is a very powerful clue that their mechanisms of action differ. Finally, extension of the alpha-methyl of hallucinogenic amphetamines to an alpha-ethyl moiety completely abolishes their hallucinogenic activity. Ultimately, we extended the alpha-methyl group of MDMA to an alpha-ethyl to afford a molecule we named (N-Methyl-1-(1,3-benzodioxol-5-yl)-2-butanamine (MBDB) that retained significant MDMA-like psychoactivity. Hence, there are three structural features that distinguish MDMA from the hallucinogenic amphetamines: (1) the *N*-methyl on the basic nitrogen, (2) the reversal of stereochemistry and, (3) tolerance of an alpha-ethyl moiety as contrasted with the alpha-methyl of hallucinogenic phenethylamines. Clearly, MDMA is distinct from classical hallucinogenic phenethylamines in its structure, and its psychopharmacology is also unique. Thus, in 1986 I proposed the name “Entactogen” for the pharmacological class of drugs that includes 3,4-methylenedioxymethamphetamine (MDMA) and other substances with a similar psychopharmacological effect. The name is derived from roots that indicate that entactogens produce a “touching within.” Rather than having significant psychostimulant, or hallucinogenic effects, MDMA powerfully promotes affiliative social behavior, has acute anxiolytic effects, and can lead to profound states of introspection and personal reflection. Its mechanism of action is now established as involving transport of MDMA by the neuronal serotonin reuptake carrier followed by carrier-mediated release of stored neuronal serotonin.

## Introduction

By the mid-1980s methylenedioxymethamphetamine (MDMA) had become a very popular recreational drug in the United States, as well as in various other countries, being widely used at all-night dance parties (raves). In the fall of 1984, I had been invited to a conference at the Esalen Institute, where a number of therapists were in attendance who had been using MDMA as an adjunct to their psychotherapy practice. I had largely been unaware of the use of MDMA by therapists and had primarily heard of its recreational use at parties and raves. The anecdotal reports offered by the conferees of the powerful effect of MDMA in therapy were eye-opening for me. Although their studies were not randomized and placebo-controlled but were only anecdotal, a typical report by a therapist who had been practicing for decades would go something like this, “this woman had been under my care for 10 years, and progress in her analysis had come to a standstill. But after being given MDMA, she suddenly opened up about traumatic events in her childhood. It was a breakthrough moment and since then we have made tremendous progress.” Such statements sounded rather unbelievable but the therapists who had been using MDMA in their practice seemed honest and sincere.

Also in attendance at this 1984 conference was Rick Doblin, a young student at New College. He had very ambitious plans to make MDMA into a medicine. At the time he had no professional credentials but was full of enthusiasm. I will come back to Rick later.

Although I had been convinced in 1984 that MDMA might have clinical utility, it seemed very doubtful that it would ever be commercialized because, in 1985, the Drug Enforcement Administration (DEA) proposed to use their emergency scheduling power to classify MDMA as a Schedule 1 controlled substance. A DEA spokesmen referred to MDMA as “just another hallucinogenic amphetamine.” Hallucinogenic amphetamines had already been relegated to Schedule 1 of the 1970 Controlled Substances Act (CSA), so logically, another “hallucinogenic amphetamine” would fall into that same category. The DEA justified the need for scheduling as related to the potential danger of MDMA use. They cited unpublished studies by Louis Seiden at the University of Chicago, who had demonstrated the loss of serotonin neurons in rodents given very high doses of MDMA (work published 2 years later by Commins et al. (1). Thus, they reasoned, the possibility of brain neuronal damage caused by MDMA use was so alarming that the drug should be immediately controlled. One could assume that MDMA would probably be relegated to the dustbin of history along with all of the other “hallucinogens” that fell into Schedule 1 of the CSA.

What I had heard at the Esalen conference, however, convinced me that MDMA had unique properties that put it outside of the structure-activity relationships known for hallucinogenic amphetamines with which I was very well versed. Earlier I also had become personally aware of the unique psychopharmacology of MDMA (2). I considered approaches that might prevent regulation of MDMA and allow its medical potential to be evaluated. My thinking was to prove that MDMA was not simply a hallucinogenic amphetamine. Here was where my expertise in the medicinal chemistry of small molecules and stereochemistry became important.

## Stereochemical and Structural Arguments for a Novel Drug Class

As we all know, every protein and peptide in our body, including receptors and transporters, is composed of L amino acids and is therefore chiral. Because of the chirality of these potential biological targets, it is a well-accepted tenet of pharmacology that if similar biologically active molecules have opposite stereochemistry, they almost certainly have different mechanisms of action.

To begin, the stereochemistry of the more active enantiomer of hallucinogenic amphetamines possesses the *R* absolute configuration at the alpha-carbon atom (3–6). Furthermore, as a graduate student I had discovered a simple way to prepare the enantiomers of substituted amphetamines (7) which made them more readily accessible for study. We used that method to prepare the enantiomers of MDMA and employed them to demonstrate that the clinical effects of MDMA resided largely in its *S*-(+) enantiomer (8).

**FIGURE 1:**
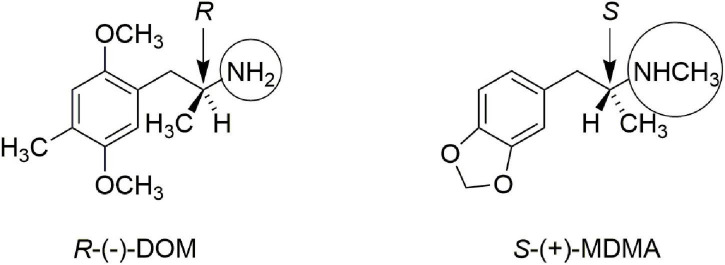
Comparison of the more active enantiomers of DOM and MDMA. Note the opposite stereochemistry at the alpha-carbon atom. Note also that DOM has a simple primary amine (circled) whereas MDMA is a secondary N-methylamine (also circled).

In addition, Lyon et al. (9) showed that the *in vitro* affinity of (+)-MDMA for [^3^H]ketanserin-labeled rat brain 5-hydroxytryptamine (serotonin, 5-HT2) receptors was only about 16 micromolar, whereas the affinity of (−)-MDMA was about five-fold higher, at 3.3 micromolar. These authors suggested it was unlikely that the effect of MDMA could be attributed to a direct 5-HT2-mediated mechanism of action. Figure 1 illustrates the stereochemistry of the more active enantiomer of the classic psychedelic agent known as DOM. Also shown in Figure 1 is the more active enantiomer of MDMA. The reader will note that the stereochemistry of the more active enantiomer of *S*-(+)-MDMA is identical that that of the more active enantiomers of amphetamine or methamphetamine. From Figure 1 one can appreciate that the more active enantiomers of DOM and MDMA have opposite stereochemistry at their α-chiral carbon atoms. The divergent stereochemistries of these two molecules is strong evidence that they must have different mechanisms of action.

Also highlighted in Figure 1 by circles is the fact that DOM, and other classic phenethylamine psychedelics, are primary amines. *N*-methylation abolishes the psychedelic activity of phenethylamines (10), yet it is a structural feature of MDMA. Thus, both the stereochemistry at the α-carbon atom, as well as the secondary methylamine of MDMA provide strong arguments that MDMA is not simply another “hallucinogenic amphetamine.”

**FIGURE 2:**
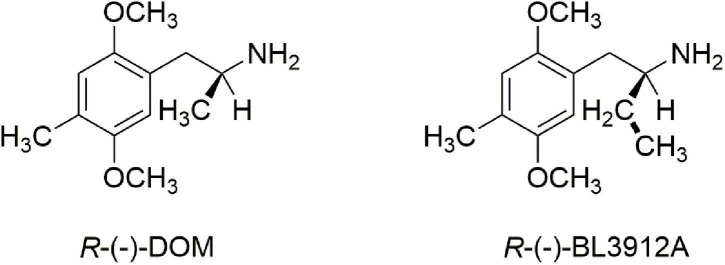
A comparison of *R*-(–)-DOM with its inactive α-ethyl analog, *R*-(–)-BL3912A.

Finally, there was a third structural feature that was worth examination. It had been demonstrated that extending the α-methyl of DOM to an α-ethyl dramatically attenuated or even abolished hallucinogenic activity (11, 12). Indeed, the *R*-(−) enantiomer of the α-ethyl congener of DOM, BL3912A (Figure 2), was taken into clinical trials and found to have no significant CNS activity at doses up to 270 mg (13), whereas DOM is orally active in the 5–10 mg range.

**FIGURE 3:**
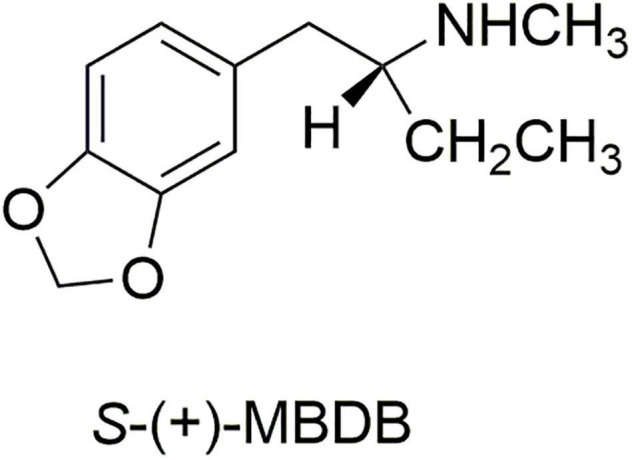
The structure of the more active enantiomer *S*-(+)-MBDB.

We therefore prepared the enantiomers of the α-ethyl analog of MDMA, which we called (N-Methyl-1-(1,3-benzodioxol-5-yl)-2-butanamine (MBDB) (14) (Figure 3). Although we believed that the α-ethyl moiety would abolish any hallucinogenic activity, we had no foreknowledge of its effect on MDMA-like psychopharmacology. We first examined the discriminative stimulus properties of the enantiomers of MDMA and MBDB in rats trained to discriminate Lysergic acid-N,N-diethylamide (LSD) (0.08 mg/kg) from saline. As anticipated, neither isomer of MDMA substituted in LSD-trained rats. Similarly, neither racemic MBDB nor either of its enantiomers substituted for the LSD stimulus. [*Somewhat later we carried out two lever drug discrimination studies in rats trained to discriminate MDMA, where MBDB fully substituted, and in* (+)*-MBDB-trained rats, where MDMA fully substituted, ultimately demonstrating symmetrical substitution between the two drugs* (15)].

At about the same time the Shulgin group carried out an uncontrolled clinical study in 14 subjects, using methods described by Shulgin et al. (16). Oral doses between 150 and 200 mg of the racemic hydrochloride salt of MBDB were administered to the subjects, all of whom had prior experience with a range of psychotropic drugs, including MDMA. No hallucinogenic effects were experienced, and there was a general consensus among the subjects that MBDB had effects very similar to MDMA, with certain differences. Specifically, the onset of action for MBDB was slower and more subtle than with MDMA, and also there was less euphoria and CNS stimulation with MBDB than with MDMA (14).

In a separate double-blind experiment, four subjects were administered either a lactose placebo, or 125 mg of the hydrochloride of either the *R* or *S* enantiomer of MBDB. Subjects could readily distinguish between the two enantiomers and found the *S*-(+)-enantiomer to be active.

Based on our stereochemical and steric arguments, as well as the anecdotal reports of users, we proposed that MDMA and compounds with a related psychopharmacology should be considered in a new drug classification. The late Ralph Metzner also reached a similar conclusion and proposed to call MDMA an “empathogen.” We found that term undesirable for several reasons. First, when considering their potential use as aids to psychotherapy, we did not envision that it was empathy in the patient for the therapist that was the goal. Empathy *for* the patient, however, is an emotion that should be expressed naturally by a competent therapist, yet the therapist is not the one who takes the medicine. Perhaps more important, however, is that these substances do not simply evoke empathy, although recent studies document that MDMA does enhance empathy (17, 18). Rather, discussion with therapists indicated that MDMA allowed subjects to retrieve repressed and often traumatic memories. If these elements were normally not available for conscious processing, MDMA must have allowed the subjects somehow to have gone within their own psyche to retrieve these events. Thus, MDMA seemed to allow or facilitate a process of internal retrieval. Therefore, we proposed the word “entactogen” as a category for this type of psychoactive drug. The word is composed of the Greek roots en and gen meaning *within* and *to generate*, respectively, and *tactus*, the Latin root for touching. Hence, entactogens are substances that allow or promote a touching within or reaching inside to retrieve repressed memories.

### Pharmacological Mechanisms Consistent With a Novel Drug Class

If we had identified a new pharmacological class, it was crucial to define its mechanism of action as distinct from other presently known classes of psychoactive agents. Although the structural and stereochemical arguments were solid, what were the implications for the pharmacology? This brief review will make no attempt to summarize the vast literature on the pharmacology of MDMA; as of December 2021, a search of the literature in PubMed with MDMA as a title word returned 1908 hits! Rather, this short review will touch only on a few key publications from around the time that we first proposed that MDMA was in a new category of psychoactive substance. That history will be followed by a few selected later reports that validated certain aspects of entactogen pharmacology.

Early studies by Cheng et al. (19) in superfused vascular strips of dog dorsal metatarsal vein had found that substituted hallucinogenic amphetamines could possess either a direct or an indirect mechanism of action. Specifically, 4-methoxyamphetamine, and 2,4- and 3,4-dimethoxyamphetamine produced tissue contractions indirectly by releasing norepinephrine from sympathetic nerve terminals, whereas 2,5-dimethoxyamphetamine elicited muscle contractions by directly stimulating α-adrenergic receptors. The enantiomers of both DOM and its 4-bromo congener DOB caused tissue contraction by directly activating 5-HT receptors. Compounds tested with the 2,5-dimethoxy aryl substitution (e.g., DOM and DOB) had only a direct action and did not cause the indirect release of neuronal transmitters in dog vascular strips. This proposal was consistent with a later study by Glennon et al. (20) where it was demonstrated that the hallucinogenic amphetamines had a direct stimulating effect on serotonin 5-HT2 receptors.

Our earliest study specifically of MDMA focused on its ability to release [^3^H]-serotonin from prelabeled rat whole brain synaptosomes (21). We found the rate of release (K min^–1^ × 10^4^) of neurotransmitter for 1.0 micromolar (+)- and (−)-MDMA was 284 ± 16 and 173 ± 6, respectively. Clearly, MDMA was a potent releaser of neuronal serotonin, with the *S*-(+)-isomer being more active. This pharmacology was distinct from the direct stimulation of 5-HT receptors by 2,5-dimethoxy-substituted amphetamines and was consistent with the anecdotal human studies cited earlier.

Battaglia et al. (22) examined racemic MDMA at a variety of brain recognition sites and reported that it had highest affinity at [^3^H]paroxetine-labeled serotonin uptake sites (610 nM). Its next highest affinities were at α_2_-adrenergic sites (3.6 μM) and 5-HT2 sites (5.1 μM), clearly implicating serotonin uptake sites as the most likely target for MDMA.

Steele et al. (23) examined the ability of the enantiomers of amphetamine, MDA, MDMA, MBDB, and DOM to inhibit the uptake of [^3^H]-monoamines into rat brain synaptosomes. As shown in Table 1, uptake inhibition was greater for all of the *S*-(+) isomers, with MDMA and MBDB having identical 5-HT uptake inhibition potency. Notable, however, was the lack of activity for the classic hallucinogen DOM, which had no inhibitory activity against any of the three uptake sites. That finding was consistent with the earlier study by Cheng et al. (19), where amphetamines with a 2-methoxy substituent were not substrates for monoamine uptake carriers in neuron terminals. We speculated that the uptake proteins had evolved to exclude monoamine analogs with an ortho-oxygen (or methoxy) because they had molecular properties resembling the potent neuronal poison 6-hydroxydopamine.

**TABLE 1:** A summary of uptake inhibition by the enantiomers of amphetamine, MDA, MDMA, MBDB, and DOM at uptake sites for 5-HT, NE, and dopamine (DA) in rat brain synaptosomes (23).

	5-HT IC50 (μM)*^a^*		NE IC50 (μM)*^b^*		DA IC50 (μM)*^c^*	
Drug	*S*-(+)-	*R*-(−)-	*S*-(+)-	*R*-(−)-	*S*-(+)-	*R*-(−)-
Amphetamine	2.65 (0.15–122.0)	>5	0.07 (0.04–0.12)	0.10 (0.04–0.25)	0.38 (0.10–1.36)	2.05 (0.69–6.66)
MDA	0.49 (0.23–1.02)	1.62 (0.69–3.87)	0.27 (0.17–0.44)	0.46 (0.27–0.81)	1.96 (0.60–7.13)	>5
MDMA	0.41 (0.22–0.74)	1.73 (1.00–3.07)	0.32 (0.19–0.56)	0.81 (0.33–2.15)	4.20 (1.52–13.36)	>5
MBDB	0.41 (0.07–1.90)	1.88 (0.76–4.88)	0.64 (0.19–2.09)	2.22 (0.47–12.92)	>5	>5
DOM	>10	>10	>10	>10	>10	>10

*^a^Inhibition of [^3^H]-5-HT into rat hippocampal synaptosomes. ^b^Inhibition of [^3^H]-NE uptake into rat hypothalamic synaptosomes. ^c^Inhibition of [^3^H]-DA uptake into rat striatal synaptosomes.*

**FIGURE 4:**
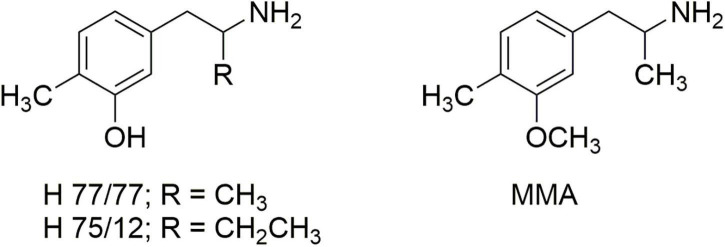
The structures of 4,α-dimethyl-meta-tyramine (H 77/77) and 4-methyl-α-ethyl-meta-tyramine (H 75/12) and 3-methoxy-4-methylamphetamine (MMA).

The studies by Steele et al. (23) seemed to contrast, however, with the report of Ho et al. (24) that removal of the ortho-methoxy of DOM afforded a compound that was nearly as potent as DOM in its ability to disrupt mouse behavior, with a comparable duration of effect. That prompted an examination of the earlier literature where we found reports by Carlsson et al. (25, 26) regarding the effects of 4,α-dimethyl-meta-tyramine (H 77/77) and 4-methyl-α-ethyl-meta-tyramine H 75/12) (Figure 4). In particular, both compounds were found to be substrates of the neuronal monoamine transporters and to release stored neuronal norepinephrine (NE) and 5-HT. In particular, the α-ethyl compound (H 75/12) was able to cause the neuronal depletion of 5-HT through a transporter-mediated process, although in 1969 the mechanism was not clearly elucidated.

Carlsson’s reports led us to examine the *O*-methyl derivative 3-methoxy-4-methylamphetamine (MMA; Figure 4) and its two stereoisomers (27). *In vitro* experiments revealed that racemic MMA was a potent and selective inhibitor of [^3^H]-5-HT uptake into rat brain synaptosomes with an IC_50_ of 138 nM, and a higher potency (IC_50_ 99 nM) for the *S*-(+)-enantiomer. Racemic MMA and both of its stereoisomers were also evaluated in the two-lever drug discrimination assay in four colonies of rats trained to discriminate saline from LSD, MDA, *S*-(+)-MBDB, or *S*-(+)-amphetamine. Racemic MMA fully substituted in MDMA-trained rats, with a potency nearly identical to that of MDMA. Both enantiomers of MMA fully substituted in *S*-(+)-MBDB-trained rats, with identical potencies of 0.46 mg/kg. Racemic MMA failed to substitute in either LSD-trained or *S*-(+)-amphetamine-trained rats. We also found in this study that, in contrast to MDMA, repeated high doses of MMA failed to produce the neuronal 5-HT deficits characteristic of MDMA.

These results were all consistent with our developing hypothesis that MDMA, MBDB, and presumably other entactogens acted primarily by a carrier-mediated release of stored neurotransmitters (i.e., serotonin and NE) from neuron terminals. Although MDMA also causes release of neuronal dopamine, the absence of that pharmacology from MBDB and its relative lack of psychostimulant effects suggested that the role of dopamine was less important to the pharmacology of MDMA and similar compounds.

Dimpfel et al. (28) compared *in vivo* telemetric recordings from awake rat frontal cortex, hippocampus, striatum, and reticular formation after intraperitoneal (ip) administration of the hydrochloride salts of *R-*(−)-DOB, *R*-(−)-DOI, *R*-(−)-DOM, *S*-(+)-MDMA, *S*-(+)-MBDB, and *S*-(+)-amphetamine. Frequency analysis of the field potentials revealed distinct spectral fingerprint differences between the different pharmacologies and dissimilarity between the hallucinogens and MDMA or MBDB. Virtually the same changes in power could be seen after *R*-(−)-DOM, *R*-(−)-DOB, and *R*-(−)-DOI, where there was increased power in the alpha_1_ frequency, particularly in the striatum, whereas after *S*-(+)-amphetamine, *S*-(+)-MDMA, and *S*-(+)-MBDB there was not. Similarly, nearly the same changes were observed after injection of *S*-(+)-MDMA or *S*-(+)-MBDB. The action of *S*-(+)-amphetamine was restricted mostly to power decreases in the delta and alpha_2_ frequency band. There was some similarity between *S*-(+)-amphetamine and *S*-(+)-MDMA, probably reflecting a dopamine-mediated psychostimulant component of MDMA, whereas *S*-(+)-MBDB lacked that effect. These studies lent further support to the hypothesis that the *in vivo* pharmacology of MDMA and MBDB is distinct from the classic hallucinogenic amphetamines, at least in rats.

Rudnick and Wall (29) studied the ability of MDMA and several other amphetamine derivatives, including MMA, to release serotonin from nerve terminals both *in vivo* and *in vitro*. MMA inhibited imipramine binding to serotonin transporters in platelet plasma membrane vesicles, inhibited Na^+^ gradient-driven serotonin transport into those vesicles, and also released [^3^H]serotonin from plasma membrane vesicles, apparently by a process of exchange. The half-maximal concentration for this effect was comparable to that reported for MDMA.

Nash et al. (30) then studied the ability of (+)- and (−)-MDMA to stimulate phosphatidylinositol turnover in cultured 3T3 and A9 fibroblast cells expressing the 5-HT_2*A*_ or 5-HT_2*C*_ receptors, respectively. Those authors reported that (+)-MDMA was completely inactive at the 5-HT_2*A*_ receptor, although it did produce about 60% of the maximal stimulation of serotonin at the 5-HT_2*C*_ receptor. A finding that again showed that the clinically more active (+)-MDMA had a different action than the classic hallucinogenic phenethylamines.

### Recent Validations for a Novel Class

Subsequent clinical studies confirmed early preclinical hypotheses. For example, Liechti and Vollenweider (31) pretreated human subjects with three different neuroreceptor blocking ligands: citalopram, ketanserin, or haloperidol. The SSRI citalopram markedly reduced most psychoactive effects of MDMA in humans, a finding indicating that the psychological effects of MDMA in humans primarily depend on carrier-mediated release of 5-HT, and consistent with preclinical studies. Similarly, Tancer and Johanson (32) reported that in human volunteers, treatment with fluoxetine, another selective SSRI, for at least 5 days attenuated many, but not all, of the subjective effects of MDMA. The effects of MDMA were also reduced by pretreatment with paroxetine, another SSRI (33). Further, the dual serotonin reuptake carrier (SERT)/norepinephrine reuptake carrier (NET) inhibitor duloxetine blocked MDMA-induced NE and 5-HT release in NET- and SERT-transfected cells and also prevented the acute psychoactive effects of MDMA in humans (34), confirming the importance of NE and 5-HT in the actions of MDMA.

More recently, Bershad et al. (35), in a clinical study, found that MDMA produced distinctive effects that were distinguishable from psychostimulants across several social domains. They noted that MDMA increased self-reported feelings of trust and generosity, and increased responses to social and emotionally valenced stimuli. MDMA also increased empathy and increased the social and emotional themes in spontaneous speech. Other recent studies have likewise reported that the psychoactive effects of MDMA in humans are distinct from those of amphetamine or other pure psychostimulants (36, 37).

Finally, in a very recent mouse study, Heifets et al. (38) carried out extensive experiments examining the prosocial and rewarding properties of MDMA. They were able to demonstrate that the action of MDMA at the serotonin transporter within the nucleus accumbens was necessary and sufficient for the prosocial effect of MDMA in mice. This action required the involvement of serotonin 5-HT_1*B*_ receptors. By contrast, the psychostimulant actions of MDMA involved dopaminergic pathways and were not relevant to the prosocial effects of MDMA.

Rick Doblin, who I referenced earlier, founded the Multidisciplinary Association for Psychedelic Studies (MAPS) in 1986, which has just completed a Phase 3 clinical trial using MDMA-assisted therapy to treat PTSD. The therapeutic effect was robust, durable, and unprecedented (39). A second Phase 3 study is beginning that if also successful should potentially lead to FDA approval of MDMA-assisted therapy as a medical procedure for treating PTSD. It is important to emphasize here that without the perseverance and dedication of Rick Doblin, entactogens would not have found their place in medicine!

## Conclusion

Structural and stereochemical arguments were definitive in distinguishing entactogens from classic psychedelic phenethylamines. There are fewer structural elements to distinguish entactogens from psychostimulants such as methamphetamine but entactogens have clinical psychoactive properties that are clearly distinct from psychostimulants. Early *in vitro* pharmacology studies indicated that a necessary, but perhaps not sufficient component of action for entactogens was the release of neuronal serotonin, which today we recognize as resulting from a reverse transport of serotonin from the neuron terminal following uptake of the entactogen molecule through the SERT. The potential role of NE release in the action of entactogens has not been sufficiently investigated but also may play some role in the psychopharmacology of entactogens. Clinical studies have demonstrated that drugs that block the serotonin reuptake carrier can block or attenuate most of the psychoactive effects of entactogens, so we hypothesize that an action at the SERT is a necessary, but perhaps not entirely sufficient explanation for the pharmacology of entactogens. Extensive investigation of MDMA-induced social affiliation in mice has been demonstrated to result from serotonin release and stimulation of serotonin 5-HT_1*B*_ receptors in the nucleus accumbens. In essence, one can envision that entactogens are the serotonergic counterparts of psychostimulants, where the mechanism of action of entactogens involves carrier-mediated release of serotonin [and norepinephrine? (40)], whereas psychostimulants act by carrier-mediated release of catecholamines from neuronal stores.

## Author Contributions

The author confirms being the sole contributor of this work and has approved it for publication.

## Conflict of Interest

The author declares that the research was conducted in the absence of any commercial or financial relationships that could be construed as a potential conflict of interest.

## Publisher’s Note

All claims expressed in this article are solely those of the authors and do not necessarily represent those of their affiliated organizations, or those of the publisher, the editors and the reviewers. Any product that may be evaluated in this article, or claim that may be made by its manufacturer, is not guaranteed or endorsed by the publisher.
